# Propensity score-based analysis of stereotactic body radiotherapy, lobectomy and sublobar resection for stage I non-small cell lung cancer

**DOI:** 10.1093/jrr/rrac041

**Published:** 2022-07-11

**Authors:** Noriko Kishi, Yukinori Matsuo, Toshi Menju, Masatsugu Hamaji, Akiyoshi Nakakura, Hideki Hanazawa, Keiichi Takehana, Hiroshi Date, Takashi Mizowaki

**Affiliations:** Department of Radiation Oncology and Image-Applied Therapy, Graduate School of Medicine, Kyoto University, 54 Shogoin-Kawahara-cho, Sakyo-ku, Kyoto 606-8507, Japan; Department of Radiation Oncology and Image-Applied Therapy, Graduate School of Medicine, Kyoto University, 54 Shogoin-Kawahara-cho, Sakyo-ku, Kyoto 606-8507, Japan; Department of Thoracic Surgery, Graduate School of Medicine, Kyoto University, 54 Shogoin-Kawahara-cho, Sakyo-ku, Kyoto 606-8507, Japan; Department of Thoracic Surgery, Graduate School of Medicine, Kyoto University, 54 Shogoin-Kawahara-cho, Sakyo-ku, Kyoto 606-8507, Japan; Department of Biomedical Statistics and Bioinformatics, Graduate School of Medicine, Kyoto University, 54 Shogoin-Kawahara-cho, Sakyo-ku, Kyoto 606-8507, Japan; Department of Radiation Oncology and Image-Applied Therapy, Graduate School of Medicine, Kyoto University, 54 Shogoin-Kawahara-cho, Sakyo-ku, Kyoto 606-8507, Japan; Department of Radiation Oncology and Image-Applied Therapy, Graduate School of Medicine, Kyoto University, 54 Shogoin-Kawahara-cho, Sakyo-ku, Kyoto 606-8507, Japan; Department of Thoracic Surgery, Graduate School of Medicine, Kyoto University, 54 Shogoin-Kawahara-cho, Sakyo-ku, Kyoto 606-8507, Japan; Department of Radiation Oncology and Image-Applied Therapy, Graduate School of Medicine, Kyoto University, 54 Shogoin-Kawahara-cho, Sakyo-ku, Kyoto 606-8507, Japan

**Keywords:** Overall survival (OS), local recurrence (LR), distant recurrence (DR), non-lung cancer death, shared decision-making

## Abstract

We applied two propensity score-based analyses to simultaneously compare three treatment modalities—stereotactic body radiotherapy (SBRT), lobectomy, or sublobar resection (SLR)—for stage I non-small cell lung cancer (NSCLC), with the aim of clarifying the average treatment effect (ATE) and formulating a risk-adapted approach to treatment selection. A retrospective review of 823 patients aged ≥65 years who underwent SBRT, lobectomy, or SLR for stage I NSCLC was conducted. The following two analyses using machine learning-based propensity scores were performed: (i) propensity score weighting (PSW) to assess the ATE in the entire cohort, and (ii) propensity score subclassification (PSS) to evaluate treatment effects of subgroups. PSW showed no significant difference in the 5-year overall survival (OS) between SBRT and SLR (60.0% vs 61.2%; *P* = 0.70) and significant difference between SBRT and lobectomy (60.0% vs 77.6%; *P* = 0.026). Local (LR) and distant recurrence (DR) rates were significantly lower in lobectomy than in SBRT, whereas there was no significant difference between SBRT and SLR. PSS identified four subgroups with different patient characteristics: lobectomy-oriented (5-year cumulative incidences of non-lung cancer death, 7.5%), SLR-oriented (14.2%), SBRT-oriented (23.8%) and treatment-neutral subgroups (16.1%). Each subgroup showed different survival trends regarding the three treatments. The ATE of SBRT was not significantly different from that of SLR, but it was inferior to lobectomy. Four subgroups with different risks of non-lung cancer death and different survival trends for each treatment were identified. These would help decision-making for patients with stage I NSCLC.

## INTRODUCTION

The standard treatment for early-stage non-small cell lung cancer (NSCLC) is lobectomy with mediastinal lymph node dissection or systematic lymph node sampling [1]. For patients who are unable to undergo lobectomy, sublobar resection (SLR) or stereotactic body radiotherapy (SBRT) is recommended [2, 3]. A pooled analysis of two small-sample randomized controlled trials (RCTs), STARS and ROSEL, suggested a potential role of SBRT in patients with operable NSCLC [4]. However, because of a lack of strong evidence based on RCTs, whether SBRT and surgery yield comparable survival outcomes in stage I NSCLC remains controversial. Recently, the SABRTooth trial, which evaluated the feasibility of an RCT comparing SBRT with surgery in high-risk surgical patients, concluded that it is not feasible in the UK because of preexisting treatment preferences [5]. Therefore, there is a need for extracting helpful information from observational studies to compare SBRT with surgery [6, 7].

Propensity score matching (PSM) is commonly used to obtain unbiased treatment effects between surgery and SBRT from observational studies [8–10]. It estimates the average treatment effect on the treated (ATT) through the comparison of the outcomes between the matched set [11]. However, the main pitfall of PSM is that information on the treatment effect in the entire cohort (the average treatment effect [ATE]) or that in the unmatched patients is omitted [12]. In addition, PSM is highly dependent on which patients are included into the matched set. Previous PSM studies comparing the ATT between surgery and SBRT present conflicting results because of the inconsistency of the matched cohort [7]. The elucidation of treatment effects in the entire cohort and in the unmatched cohorts would provide both patients and physicians with better understanding of the treatment options. This would guide patients, especially elderly patients, in selecting a treatment with consideration of the outcomes that can be expected from SBRT and surgery.

Therefore, we conducted two types of propensity score-based analyses to compare the ATE and to formulate a risk-adapted treatment selection in patients who underwent SBRT, lobectomy, or SLR for stage I NSCLC: Propensity score weighting (PSW), which provides the ATE in the entire cohort by creating a virtual cohort where all patients are included, and propensity score subclassification (PSS), which divides patients into subgroups according to their propensity scores [12] and provides the treatment effect for the patient-specific subgroup.

## MATERIALS AND METHODS

This study was performed in accordance with the Declaration of Helsinki (1975, as revised in 2013) and was approved by the Kyoto University Ethics Committee in September 2019 (approval number, R2123). The need for written informed consent was waived because of the retrospective study design.

### Patient population

Data on patients with clinical stage I NSCLC (based on the Union for International Cancer Control 7^th^ edition), aged ≥65 years, and treated with lobectomy, SBRT or SLR because of the presence of medical comorbidities between January 2003 and February 2014 in Kyoto University Hospital were retrospectively reviewed. Before the clinical diagnosis of stage I NSCLC, chest computed tomography (CT), with or without ^18^F-fluorodeoxyglucose positron emission tomography (FDG-PET), and brain magnetic resonance imaging were performed. FDG-PET/CT was not available in our institute before 2009 and was not routinely performed for patients with a ground-glass opacity nodule. Biopsy using endobronchial ultrasound (EBUS) or mediastinoscopy was performed for patients with a lymph node suspicious of metastasis. In the absence of histological confirmation, NSCLC was clinically diagnosed based on the patient’s history, imaging findings and laboratory data by a multidisciplinary oncology team, including thoracic surgeons, pulmonologists, diagnostic radiologists and radiation oncologists. Patients who had synchronous second primary lung cancer [13] at diagnosis, who underwent intentional SLR, who had an Eastern Cooperative Oncology Group Performance Status (ECOG-PS) score ≥ 2 or unknown, or in whom the prescription dose for SBRT was < 100 Gy in a biologically effective dose at an alpha/beta ratio of 10 Gy (BED_10_) to the isocenter were excluded. Intentional SLR was defined as SLR for ground-glass opacities, which are associated with low-grade malignancy and presumably do not require a lobectomy before undergoing surgery. Each patient was assigned to a group according to the first received intervention during that period.

### Treatment procedures

Lobectomy or SLR was performed in the lateral decubitus position under general anesthesia using single-lung ventilation through a double-lumen tracheobronchial tube; in lobectomy, mediastinal lymph node dissection was performed. In almost all cases, lobectomy involved video-assisted thoracic surgery. SLR included both wedge resection and segmentectomy, and some SLR patients underwent lymph node sampling. The SBRT procedures have been previously presented [14, 15]. Briefly, the patient was immobilized with a Stereotactic Body Frame (Elekta, Stockholm, Sweden) until April 2008, and a BodyFIX (Elekta) thereafter with both arms raised. Tumor motion was assessed by X-ray fluoroscopy and if it exceeded 8 mm in the longitudinal direction, a pressure plate was used to reduce the amplitude of motion. The internal target volume was delineated based on a CT scan with a slow-scan technique until October 2006, and a four-dimensional CT scan thereafter. A 5-mm margin was added to create the planning target volume. The irradiation plan was created using treatment planning systems: Eclipse (Varian Medical Systems, Palo Alto, CA) until April 2008, and iPlan (BrainLAB, Feldkirchen, Germany) thereafter. The treatment plans were created using 5–8 multiple non-coplanar, static 6-MV photon beams from Clinac 2300 C/D (Varian Medical Systems) until October 2006, Novalis (BrainLAB) until November 2010, and Vero 4DRT (Hitachi Ltd., Tokyo, Japan) thereafter. The prescribed doses to the isocenter were 48 Gy in four fractions for peripheral tumors (BED_10_, 105.6 Gy) and 60 Gy in eight fractions for centrally located tumors (BED_10_, 105.0 Gy). In June 2009, the prescribed dose was increased to 56 Gy (BED_10_, 134.4 Gy) for peripheral tumors with a diameter of > 30 mm.

As an adjuvant therapy, a combination of uracil and tegafur was orally administered to patients with a tumor diameter of > 20 mm [16]. Platinum-based chemotherapy was administered to patients whose disease was upstaged to ≥ pathological stage II.

Follow-up visits with physical examination and chest CT or radiography were performed every 3–6 months up to the fifth year and every 6–12 months thereafter. Upon suspected recurrence, FDG-PET/CT and/or brain MRI were performed. Primary tumor recurrence after SBRT was diagnosed through histologic confirmation or observation of continuous enlargement of the local tumor on CT for at least 6 months.

There were nine pretreatment variables identified, namely, age, sex, ECOG-PS, smoking status, body mass index (BMI), Charlson comorbidity index (CCI) [17], forced expiratory volume in 1 second (FEV1), tumor diameter and C/T ratio, which is equal to the maximal diameter of consolidation divided by the maximal tumor diameter [18]. Histological data were also extracted for patients with biopsy-proven or surgical pathology. Data on survival and recurrence patterns were collected for post-treatment outcome evaluation.

Overall survival (OS) was defined as the period between the day of surgery or the initial day of SBRT and death from any cause and was censored on the last day of the follow-up. Recurrence-free survival (RFS) was defined as the period between the day of surgery or the initial day of SBRT and the date of recurrence or death and was censored on the last day of the follow-up with recurrence-free status verification. Local recurrence (LR), regional recurrence (RR) and distant recurrence (DR) were defined according to the American College of Chest Physicians and Society of Thoracic Surgeons Consensus Statement [19].

### Statistical analyses

Differences in patients’ characteristics among the three treatment groups were evaluated using the chi-square test for categorical variables and the t-test for continuous variables. The median follow-up period was estimated using the reverse Kaplan–Meier method for potential follow-up [20]. OS and RFS were calculated using the Kaplan–Meier method. The cumulative incidence rate of risk of death from causes other than lung cancer (non-lung cancer death) was calculated for each treatment group using the cumulative incidence function, accounting for lung cancer-related death as a competing risk. The cumulative incidence rates of LR, RR and DR were also calculated, with non-lung cancer death taken as a competing risk. A Cox proportional hazards model was used to evaluate the effect of treatment on the outcomes with SBRT as a reference.

The propensity scores for SBRT (PS_SBRT_), lobectomy (PS_Lob_) and SLR (PS_SLR_) in each individual were estimated using the generalized boosted model with a five-fold cross-validation performed to avoid overfitting [21]. Nine pretreatment factors that affected the treatment decision were included in the model: sex (male or female), ECOG-PS (0 or 1), smoking status (current, former or never) and BMI (< 18.5 kg/m^2^, underweight; 18.5–25 kg/m^2^, normal weight; ≥ 25 kg/m^2^, overweight) as categorical variables, and age, CCI, FEV1, tumor diameter and C/T ratio as continuous variables. According to PS_SBRT_, PS_Lob_ and PS_SLR_, each individual’s weight was calculated using marginal mean weighting with the stratification method [22]. For the PSS, thresholds for PS_SBRT_, PS_Lob_ and PS_SLR_ were determined using the maximally selected log-rank statistics for OS in patients who underwent SBRT, lobectomy and SLR, respectively.

Statistical significance was set at *P* < 0.05, except for multiple comparisons of survival outcomes or cumulative incidence of recurrence between SBRT and lobectomy and between SBRT and SLR. For each of these cases, the statistical significance was set at *P* < 0.025 after Bonferroni correction. Statistical analysis was performed using R software (version 4.0.2) with the gbm (version 2.1.8), WeightIt (version 0.10.2), and survminer (version 0.4.8) packages.

## RESULTS

### Patient characteristics

A total of 1028 stage I NSCLC patients were treated with SBRT, lobectomy, or SLR between January 2003 and February 2014. Among them, 823 patients were enrolled in the study (SBRT, 204; lobectomy, 480; SLR, 139) (Supplemental Figure 1). At the data cutoff point (January 2020), the median follow-up periods for SBRT, lobectomy and SLR were 8.9, 7.3 and 7.1 years, respectively. The clinical nodal stage was determined by FDG-PET in 684 patients (84.4%). Thirteen patients who were suspected of having N1 metastasis were proven to be cN0 by EBUS or mediastinoscopy. In the remaining 126 patients, clinical N stage was based on CT images. The proportion of patients who were men, underweight, had a prior history of smoking and ECOG-PS of 1 was higher in the SBRT group than in the lobectomy or SLR group. The tumor diameter was smaller in the SLR group than in other groups (Table 1). In the SLR group, 75 patients underwent segmentectomies, and 64 underwent wedge resections. The prescribed doses for SBRT were 48 Gy in 4 fractions (*n* = 159), 56 Gy in 4 fractions (*n* = 20), 60 Gy in 8 fractions (*n* = 23) and other fractionations (n = 2). No treatment-related death was observed in the lobectomy or SLR group, but grade 5 radiation pneumonitis occurred in one SBRT patient.

**Table 1:** Patient characteristics in the unweighted and PSW cohorts

	**Unweighted cohort**					**Weighted cohort**				
	**Overall**	**SBRT**	**Lobectomy**	**SLR**	***P* value**	**Overall**	**SBRT**	**Lobectomy**	**SLR**	***P* value**
	n = 823	*n* = 204	*n* = 480	*n* = 139		*n* = 823	n = 204	n = 480	n = 139	
Age [years]	74.6 ± 5.7	78.3 ± 5.8	72.6 ± 4.8	75.9 ± 5.2	< 0.001	74.8 ± 5.5	75.2 ± 5.1	74.7 ± 5.6	74.7 ± 5.9	0.88
Sex (Male/Female)	514/309	152/52	274/206	88/51	< 0.001	503/320	116/88	296/184	91/48	0.76
ECOG-PS (0/1)	659/164	113/91	449/31	97/42	< 0.001	651/172	164/40	370/110	117/22	0.54
Smoking status (Current/ Former/Never)	172/394/ 257	17/153/34	121/172/ 187	34/69/36	< 0.001	177/384/ 262	44/97/63	101/222/ 156	32/64/43	1.00
BMI (Under/Normal/ Overweight)	98/565/ 159	37/134/32	46/339/95	15/92/32	0.017	71/597/ 155	18/165/21	46/336/98	7/96/35	0.14
CCI (0/1-2/3+)	273/377/ 173	32/108/64	214/198/ 68	27/71/41	< 0.001	322/339/ 162	97/75/32	172/204/ 104	53/60/26	0.63
FEV1 [L]	1.99 ± 0.61	1.61 ± 0.62	2.16 ± 0.55	1.92 ± 0.54	< 0.001	2.00 ± 0.56	1.90 ± 0.51	2.02 ± 0.55	2.06 ± 0.61	0.20
Tumor diameter [mm]	24.9 ± 9.1	24.1 ± 8.1	26.4 ± 9.2	20.7 ± 8.6	< 0.001	25.4 ± 8.8	25.4 ± 7.9	24.8 ± 9.2	27.4 ± 8.4	0.10
C/T ratio	0.87 ± 0.30	0.93 ± 0.23	0.85 ± 0.31	0.87 ± 0.32	0.002	0.88 ± 0.27	0.88 ± 0.25	0.87 ± 0.28	0.91 ± 0.25	0.78
Histology Ad/Sq/Others/ Unknown	509/209/ 66/39	84/57/ 24/39	341/107/ 32/0	84/45/ 10/0	< 0.001	554/188/ 63/18	128/40 /18/18	332/114/ 34/0	94/34/ 11/0	0.007

Data are presented as mean ± standard deviation (SD) for continuous variables and as numbers for categorical variables. Data were not available for BMI (1 SBRT), FEV1 (15 SBRT, 23 lobectomy and 2 SLR), tumor diameter (5 lobectomy and 4 SLR), and C/T ratio (2 SBRT, 7 lobectomy and 4 SLR).

*Abbreviations:* Ad, adenocarcinoma; BMI, body mass index; C/T ratio, consolidation/tumor ratio; CCI, Charlson comorbidity index; ECOG-PS, Eastern Cooperative Oncology Group performance status; FEV1, forced expiratory volume in 1 second; SBRT, stereotactic body radiotherapy; SLR, sublobar resection; Sq, squamous cell carcinoma.

The number of post-surgical upstaging to ≥ pathological stage II were 86 patients following lobectomy and eight patients following SLR, respectively. Among the upstaged patients, 60 following lobectomy and three following SLR received adjuvant chemotherapy. No patients treated with SBRT received adjuvant chemotherapy.

### Unweighted original cohort

**Fig. 1:**
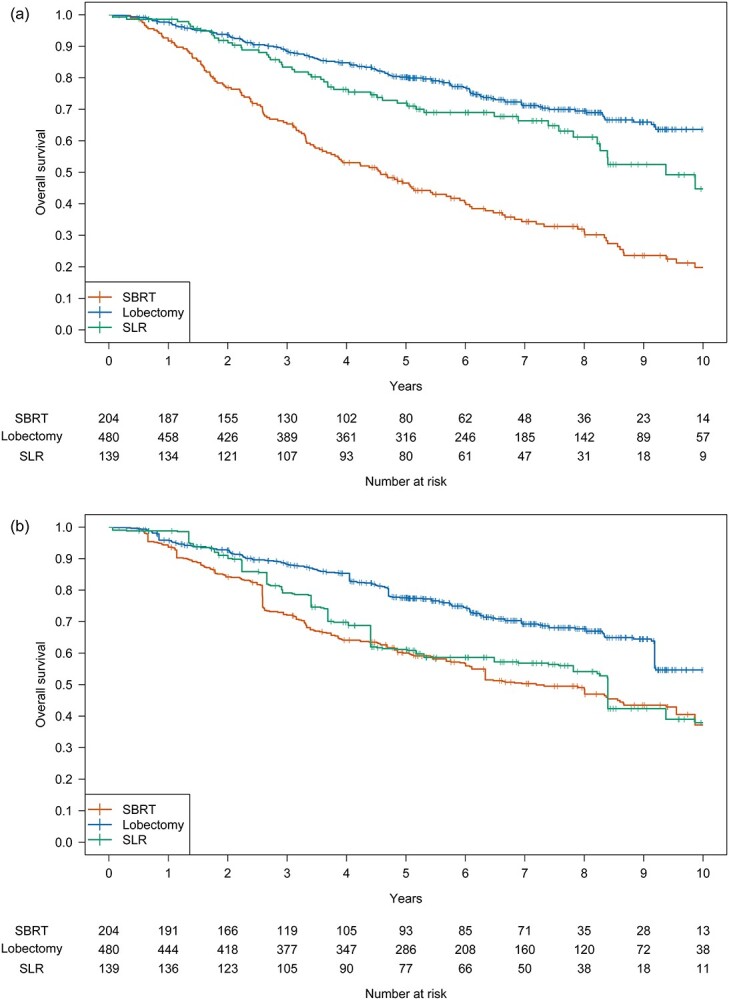
Kaplan–Meier curves for OS (a) in the unweighted cohort and (b) in the PSW cohort.

**Fig. 2:**
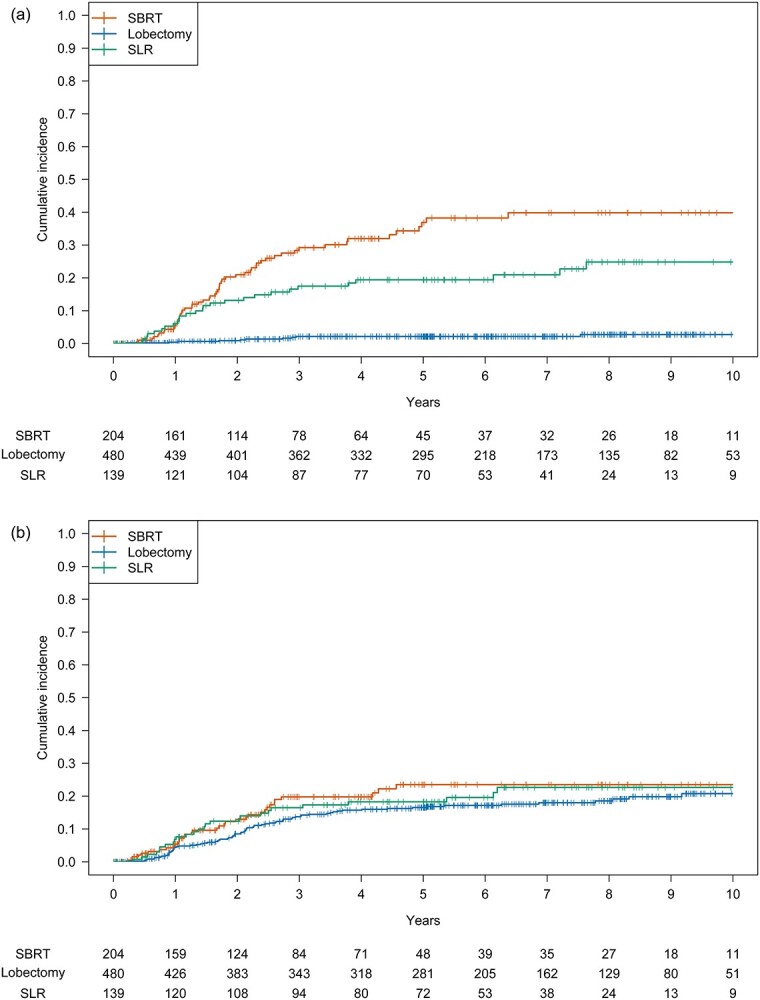

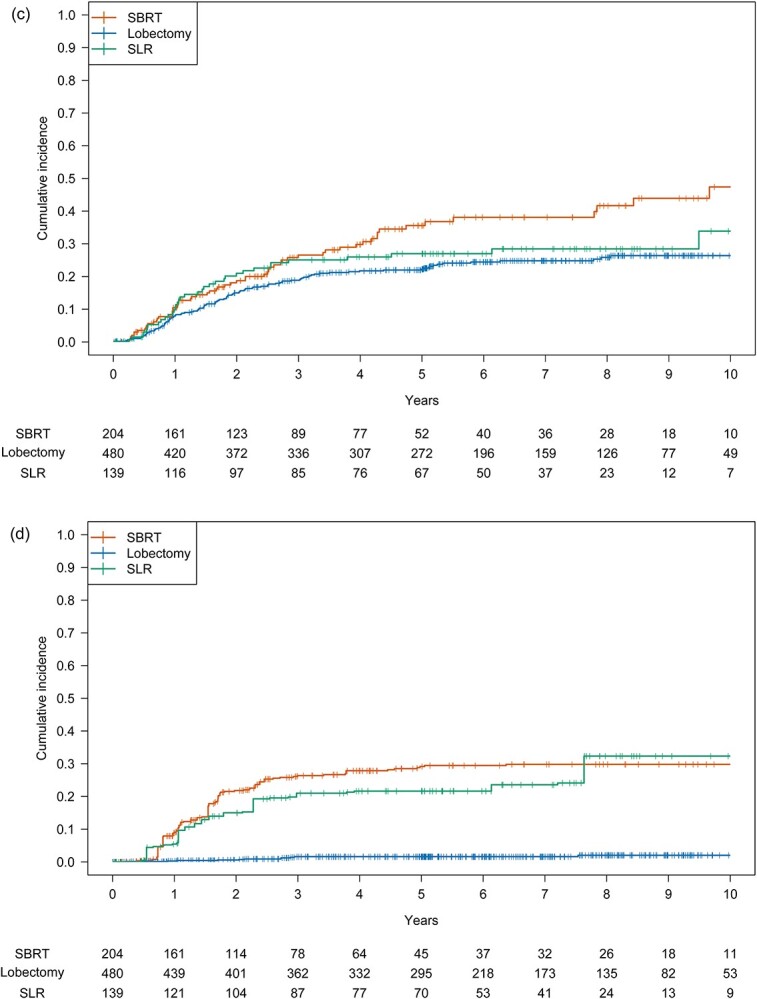

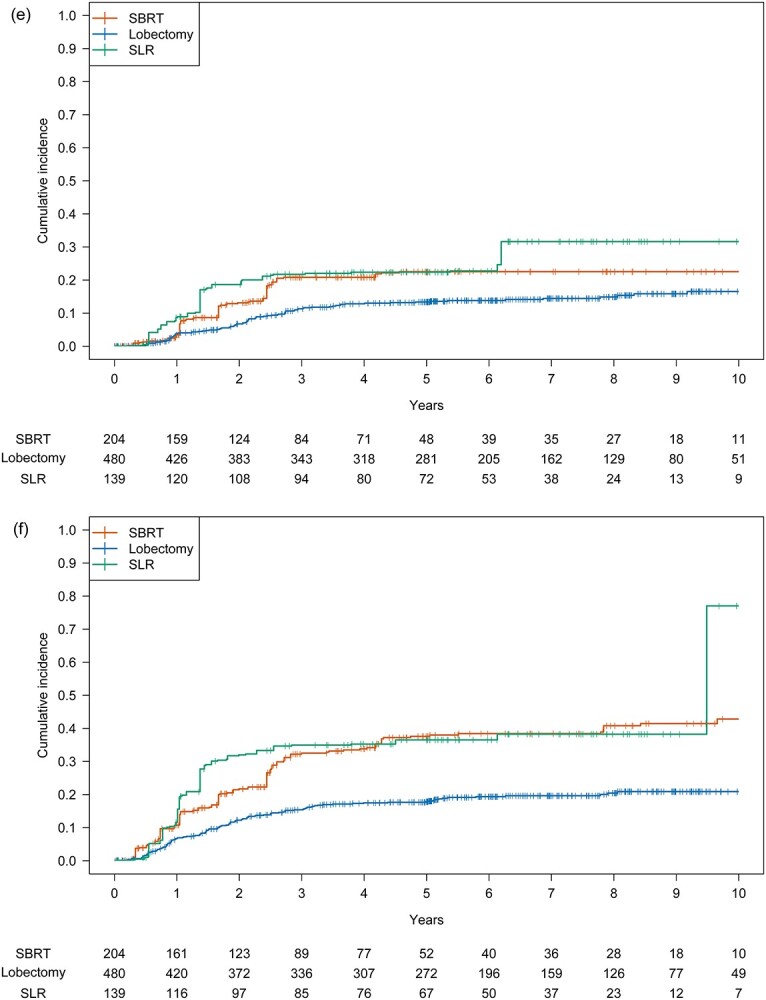
Cumulative incidence rates of LR, RR and DR in the unweighted cohort (a–c) and in the PSW cohort (d–f).

The 5-year OS and RFS rates of the SBRT, lobectomy and SLR groups were 46.6%, 80.2% and 71.9%, respectively, and 31.1%, 67.0% and 56.9%, respectively. The OS of the SBRT group was significantly lower than those of the lobectomy and the SLR groups (hazard ratio [HR] for lobectomy, 0.30; 97.5% confidence interval [CI], 0.23–0.40; *P* < 0.001; HR for SLR, 0.43; 97.5% CI, 0.30–0.62; *P* < 0.001) (Fig. 1a). The RFS was significantly lower than that of SBRT (HR for lobectomy, 0.36; 97.5% CI, 0.28–0.46; *P* < 0.001; HR for SLR, 0.54; 97.5% CI, 0.39–0.75; *P* < 0.001) (Supplemental Figure 2). The cumulative incidence of non-lung cancer death was significantly higher following SBRT than following lobectomy and SLR (Supplemental Figure 3, Supplemental Table 1). The cumulative incidences of LR and DR of SBRT were significantly higher than those of lobectomy and SLR. No significant difference was found in the incidence of RR (Figs 2a–c, Supplemental Table 1).

### Propensity score weighted cohort

**Fig. 3:**
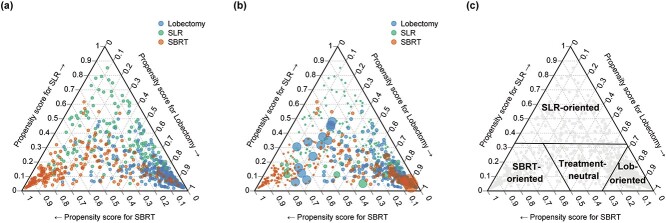
Ternary plots of propensity scores for SBRT, lobectomy and SLR in the original unweighted cohort (a) and in the PSW cohort (b); thresholds for classification of the treatment-oriented subgroups (c). Each dot indicates an individual patient with the three propensity scores, and its color indicates the selected treatment (vermillion for SBRT, blue for lobectomy and green for SLR). The size of the dot represents the weight assigned to the individual.

The relative influences of nine covariates for the propensity score estimation were as follows, in descending order: FEV1 (31.7), tumor diameter (27.6), age (16.3), CCI (6.7), smoking history (5.3), ECOG-PS (5.1), BMI (2.9), C/T ratio (2.9) and sex (1.5). The distributions of PS_SBRT_, PS_Lob_ and PS_SLR_ were presented in Supplemental Figure 4. The ranges of the assigned weights to SBRT, lobectomy and SLR patients were 0.35–25.5, 0.61–9.2 and 0.34–8.7, respectively (Figs 3a–b). In the weighted cohort, there was no significant difference in the nine covariates among the three treatments (Table 1). Our machine-learning model for the estimation of propensity scores is available at https://radonc-kyoto.shinyapps.io/psestimator/.

The 5-year OS and RFS of SBRT, lobectomy and SLR were 60.0%, 77.6% and 61.2%, respectively (Fig. 1b), and 45.1%, 66.8% and 41.9%, respectively (Supplemental Figure 2). Lobectomy tended to have a better OS than SBRT, but the difference in RFS was not significant (HR for OS, 0.54; 97.5% CI, 0.29–1.00; *P* = 0.026; HR for RFS, 0.53; 97.5% CI, 0.29–0.97; *P* = 0.018). The difference between SBRT and SLR was not significant (HR for OS, 0.87; 97.5% CI, 0.40–1.92; *P* = 0.70; HR for RFS, 1.15; 97.5% CI, 0.55–2.38; *P* = 0.67).

The cumulative incidence of non-lung cancer death was not significantly different among the three treatments (Supplemental Figure 3, Supplemental Table 1). The cumulative incidences of LR and DR were significantly lower following lobectomy than following SBRT; the difference between SBRT and SLR was not significant (Figs 2d–f, Supplemental Table 1). Finally, the cumulative incidence of RR was not significantly different among the three groups.

### Propensity score subclassification

For the subclassification, the PS_SBRT_, PS_Lob_ and PS_SLR_ thresholds were calculated as 0.50, 0.69 and 0.33, respectively. According to these thresholds, the unweighted cohort was divided into four subgroups: lobectomy-oriented (PS_Lob_ ≥ 0.69), SLR-oriented (PS_Lob_ < 0.69 and PS_SLR_ ≥ 0.33), SBRT-oriented (PS_SBRT_ ≥ 0.50 and PS_SLR_ < 0.33) and remaining treatment-neutral groups (PS_SBRT_ < 0.50, PS_Lob_ < 0.69 and PS_SLR_ < 0.33; Fig. 3c).

Different patient characteristics were identified among the four subgroups (Table 2), and the details according to each treatment are shown in Supplemental Table 2. The 5-year cumulative incidences of non-lung cancer death were 7.5%, 14.2%, 23.8% and 16.1% in the lobectomy-oriented, SLR-oriented, SBRT-oriented and treatment-neutral groups, respectively.

**Table 2:** Patient characteristics in each subgroup

	**Lobectomy-oriented**	**SLR-oriented**	**SBRT-oriented**	**Treatment-neutral**	***P* value**
	*n* = 407	*n* = 101	*n* = 150	*n* = 165	
Age [years]	71.6 ± 4.1	76.5 ± 4.9	80.0 ± 5.6	75.8 ± 4.8	< 0.001
Sex (Male /Female)	223/184	65/36	113/37	113/52	< 0.001
ECOG-PS (0/1)	405/2	66/35	64/86	124/41	< 0.001
Smoking status (Current/Former/Never)	107/124/176	26/51/24	9/121/20	30/98/37	< 0.001
BMI (Under/Normal/Overweight)	33/290/84	10/65/26	28/99/23	27/111/26	0.003
CCI (0/1–2/3+)	214/154/39	13/49/39	17/85/48	29/89/47	< 0.001
FEV1 [L]	2.23 ± 0.55	1.85 ± 0.45	1.40 ± 0.54	1.96 ± 0.46	< 0.001
Tumor diameter [mm]	27.0 ± 8.8	15.3 ± 4.2	25.0 ± 7.8	25.3 ± 9.3	< 0.001
C/T ratio	0.83 ± 0.32	0.89 ± 0.31	0.95 ± 0.21	0.89 ± 0.28	0.001
Histology (Ad/Sq/Others/Unknown)	299/80/28/0	62/29/7/3	60/43/18/29	88/57/13/7	< 0.001

Data are presented as mean ± SD for continuous variables and as numbers for categorical variables.

Abbreviations are the same as Table 1.

**Fig. 4:**
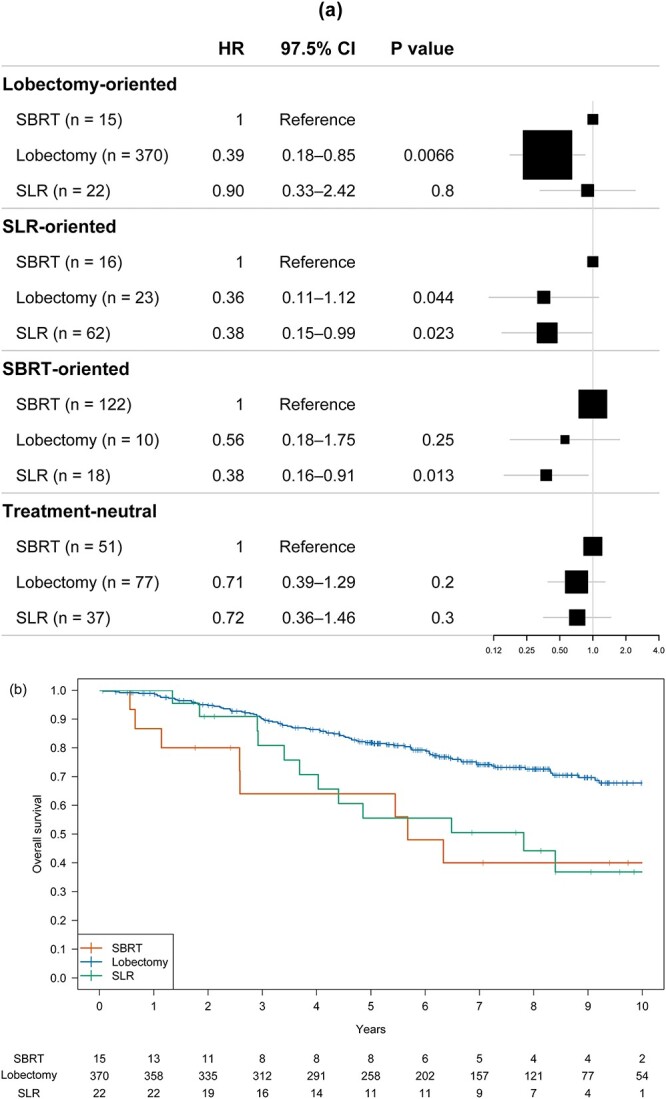

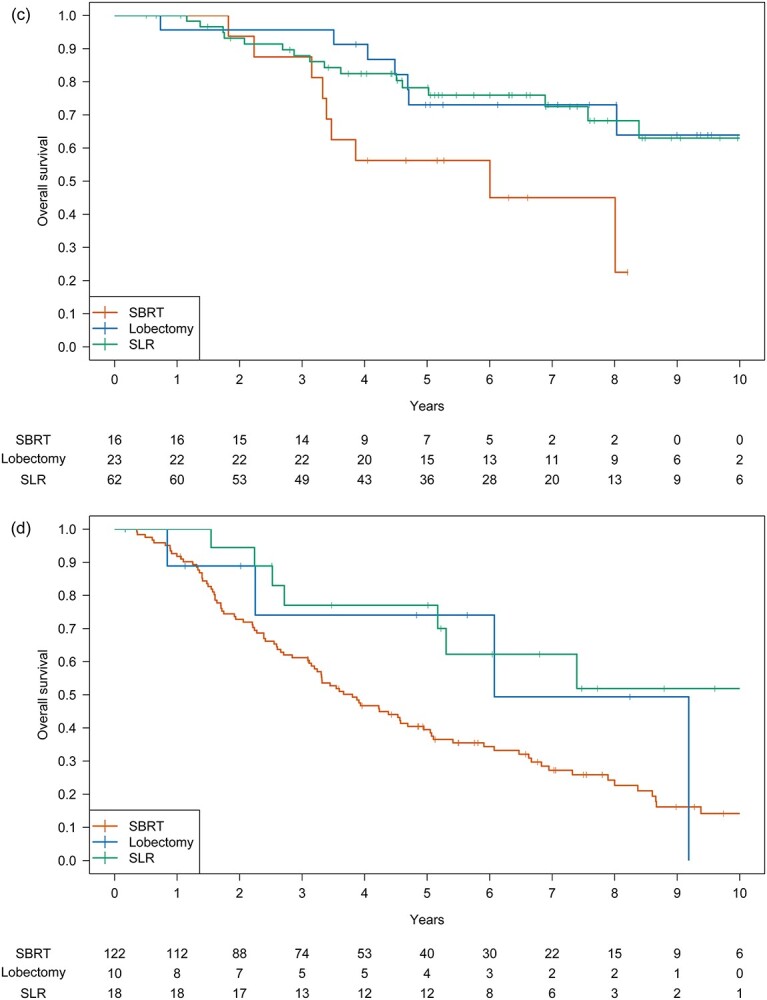

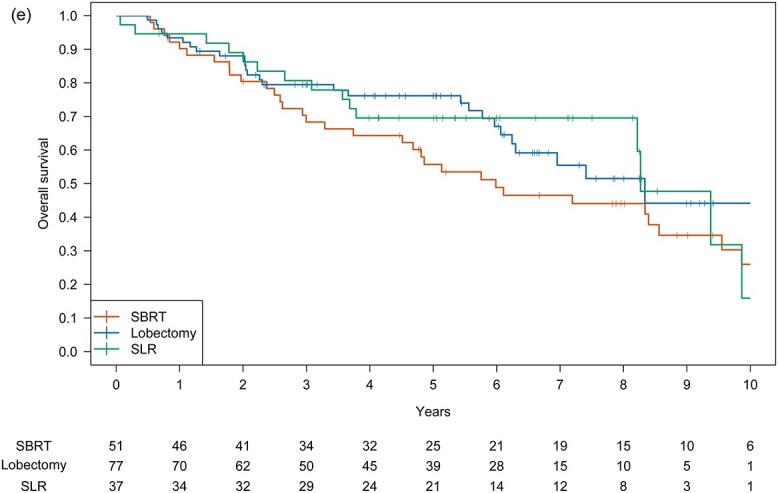
Forest plots (a) and Kaplan–Meier curves (b–e) of OS for the lobectomy-oriented, SLR-oriented, SBRT-oriented and treatment-neutral subgroups. The size of the square represents the number of patients.

Each subgroup showed different survival trends among the three treatments (Fig. 4). In the lobectomy-oriented group, OS with lobectomy was significantly higher than that with SBRT (*P* = 0.007), whereas the difference between SBRT and SLR was not significant (*P* = 0.80). In the SLR-oriented group, OS with both lobectomy and SLR tended to be better than that with SBRT (*P* = 0.044 and *P* = 0.023, respectively). In the SBRT-oriented group, the difference in OS between SBRT and lobectomy was not significant (*P* = 0.25), but the difference in OS between SBRT and SLR was significant (*P* = 0.013). In the treatment-neutral group, OS with SBRT did not differ significantly from OS with lobectomy and SLR (*P* = 0.20 and *P* = 0.30, respectively).

## DISCUSSION

The simultaneous comparison of the three treatment modalities using the PSW method revealed that the ATE of SBRT was inferior to lobectomy, but it was not significantly different from SLR. The subclassification identified four subgroups with different risks of non-lung cancer death and different survival trends for each treatment. Based on these results, a multidisciplinary oncology team will be able to provide an appropriate treatment option for patients considering the risk of non-lung cancer death in addition to the operative risk. Patients will also be able to consider the outcomes that could be expected from their preferred treatments and from treatments other than the most ‘probable’ treatment determined by the team.

The PSW analysis suggested that the difference in OS among lobectomy, SLR and SBRT is attributed to the difference in lung cancer death. Lobectomy showed significantly lower incidences of LR and DR, which might contribute to its lower lung cancer death that those of SLR and SBRT. As for distant control, the advantage of lobectomy is that precise pathological examination of lymph nodes would lead to adjuvant therapy. In the present study, post-surgical upstaging to pathological stage ≥II was observed in 86 (18%) of 480 lobectomy patients, and in 60 (70%) patients who received adjuvant chemotherapy. However, considering that a meta-analysis showed that the improvement in 5-year OS was only 4% with adjuvant therapy in resected stage I–IV NSCLC [23], it is unlikely that the administration of adjuvant therapy can fully explain the reduction in lung cancer death in lobectomy in the present study. The low incidence of LR in lobectomy might have directly contributed to the decrease of DR, as shown in breast and rectal cancer [24, 25].

In addition to the initial treatment, we should also consider the treatment at recurrence, which is determined by the general condition of patients, site of recurrence and histology. Recently, systemic therapy for NSCLC has been rapidly evolving, and subclassification with histology or driver mutations is crucial for choosing cytotoxic drugs, targeted therapy, or immunotherapy. The benefit of surgery over SBRT is the availability of pathological diagnosis with sufficient materials. In the SBRT patients of this study, the histology is unknown in 19% of the patients, and they might have missed a chance to receive targeted therapy or immunotherapy at recurrence. Moreover, the differences in the patients’ values and preferences between surgery and SBRT may have contributed to the choice of treatment at recurrence. Sullivan *et al.* reported the patients’ values by asking those treated with surgery or SBRT about the most relevant factor to their health decisions [26]. In both patient groups, the highest weighted value was the ability to stay at their own home. The second-highest weighted value was curability for patients treated with surgery, whereas it was the ability to take care of themselves for patients with SBRT. These preferences resulted in a low rate of post-recurrence treatment in SBRT patients.

The results from PSS suggested that our treatment decision was mainly based on the risk of non-lung cancer death, which ranged from 7.5% at 5 years in the lobectomy-oriented group to 23.8% in the SBRT-oriented group. When determining treatment options for stage I NSCLC patients, the multidisciplinary oncology team assesses ‘operability,’ considering factors such as pulmonary function, tumor diameter and age. At the same time, the team evaluates the balance between the potential risk of non-lung cancer death and the treatment burden. Our results validated the treatment selection algorithm by quantifying the factors associated with operability and non-lung cancer death as propensity score. In the lobectomy-oriented group, lobectomy was preferred as the guidelines recommend. In the SLR-oriented group, where the risk of non-lung cancer death was higher than that of the lobectomy-oriented group, surgical resection, including SLR and lobectomy, was preferred. In the treatment-neutral group, the risk of non-lung cancer death was between the SLR-oriented group and the SBRT-oriented group. The combined risk of lung cancer death and non-lung cancer death was similar among the three treatments. Thus, any of the three treatments could be considered as an optimal treatment. In the SBRT-oriented group with the highest risk of non-lung cancer death, SBRT was provided for most of the patients as a less invasive treatment. However, surgery showed better OS than SBRT. This might suggest two possibilities: that surgery is beneficial for some of the patients in this group and that the nine covariates using for the propensity score estimation were insufficient to evaluate operability and non-lung cancer death. This study did not include dialysis dependency or the mortality rate for comorbid malignancies [27, 28].

Decision-making for stage I NSCLC is largely influenced by physicians’ preferences and how they weigh available scientific evidence [29–31]. Meanwhile, during decision-making processes, 74% of patients feel insufficiently involved [32]. Our results provide physicians and patients with appropriate information on where the patient stands in the entire population and what survival outcomes could be expected from each treatment option. Our findings can help both physicians and patients in the decision-making process for treatment selection. For example, a 75-year-old man with ECOG-PS of 0, previous history of smoking, BMI of 18, CCI of 2, FEV1 of 2.1, tumor diameter of 2 cm and C/T ratio of 1 is classified as the treatment-neutral subgroup according to our machine-learning model for the estimation of propensity scores. If the patient refuses surgery but is concerned about the outcomes of SBRT, physicians could explain that the results of that treatment would be similar with those in the treatment-neutral subgroup, respecting the patient’s preferences and values.

This study has some limitations. First, propensity score methods have inherent biases associated with the inability to adjust for unmeasured covariates, such as tumor location (central or peripheral), which is important when deciding surgical procedure or dose prescription in SBRT. As the PSW approach is known to be at risk of an extreme weight, we applied the marginal mean weighting with the stratification method to PSW to avoid an extreme weight. This method stratifies the cohort based on the propensity score for each treatment, followed by the assignment of a weight to each stratum. Second, this is a retrospective, single-center study with a small sample size. Because the external validity of our propensity model has not yet been assessed, caution needs to be taken when using the model for future patients. Validation studies using prospective data from multiple centers are warranted. Third, this study does not reflect recent advances in NSCLC treatment because the eligible patients were treated between 2003 and 2014. Regarding SBRT, the dose regimen in this study is considered suboptimal in current clinical practice. In 2007, Onishi *et al.* showed that BED_10_ > 100 Gy to the isocenter correlated with improved local control and survival [33]; this was adopted as our eligibility criterion. However, a recent study showed that BED_10_ ≥ 130 Gy may confer an additional survival benefit [34]. An ongoing phase III RCT investigating the superiority of local dose escalation to the tumor may provide clarity on the survival benefit [35]. In terms of surgery, patients who underwent intentional SLR were excluded from the analysis of this study. Recently, JCOG 0802/WJOG 4607 L, a randomized phase III trial comparing segmentectomy with lobectomy for a peripheral tumor ≤ 2 cm with C/T ratio > 0.5, showed the benefits of segmentectomy in OS and FEV1 [36]. A model for treatment selection needs to be constantly updated to reflect these treatment advances.

In conclusion, when comparing the three treatment modalities simultaneously, the ATE of SBRT was not significantly different from that of SLR, but it was inferior to lobectomy. Four subgroups were identified with different risks of non-lung cancer death, which showed different survival trends for each treatment. Our findings would help decision-making for patients with stage I NSCLC by offering the overall view of the outcomes among the three treatments. Furthermore, these findings can provide both physicians and patients a general understanding of each treatment option.

## CONFLICT OF INTEREST

Toshi Menju, Masatsugu Hamaji, Akiyoshi Nakakura, Hideki Hanazawa, Keiichi Takehana, Hiroshi Date have nothing to disclose; Yukinori Matsuo received a research grant from Varian Medical Systems and payment for lectures from AstraZeneca K.K. outside the submitted work; Noriko Kishi received payment for presentations from Varian Medical Systems and AstraZeneca K.K. outside the submitted work; Takashi Mizowaki received funding from the Japan Agency for Medical Research and Development which partly supported English editing for the present manuscript. He has been involved in a commissioned project of Varian Medical Systems, has received advisement fee and payment for lectures from Varian Medical Systems, scholarship donation and payment for lectures from Hitachi Ltd., payment for lectures from Elekta, and payment for lectures from Brainlab, and have a contract for academic guidance on Brainlab AG outside the submitted work.

## FUNDING

This work was supported by the Japan Agency for Medical Research and Development (AMED) [grant number JP20ck0106626].

## AUTHOR STATEMENT

Noriko Kishi: data curation, formal analysis, investigation, software, visualization and writing the original draft; Yukinori Matsuo: conceptualization, project administration, methodology, formal analysis, software, and writing, reviewing and editing; Toshi Menju: resources and writing, reviewing and editing; Masatsugu Hamaji: resources and writing, reviewing and editing; Akiyoshi Nakakura: formal analysis, validation, and writing, reviewing and editing; Hideki Hanazawa: writing, reviewing and editing; Keiichi Takehana: writing, reviewing and editing; Hiroshi Date: supervision and writing, reviewing and editing; Takashi Mizowaki: supervision, funding acquisition, and writing, reviewing and editing.

## Supplementary Material

Supplementary_files_20220515fin_rrac041
