# Supplementary material for: Propensity score-based analysis of stereotactic body radiotherapy, lobectomy and sublobar resection for stage I non-small cell lung cancer
Source: J Radiat Res. 2022 Jul 11;63(5):758–71. doi: 10.1093/jrr/rrac041 (PMC9494527; doi:10.1093/jrr/rrac041)
Supplement: Supplementary_files_20220515fin_rrac041 [file supplementary_files_20220515fin_rrac041.docx]

**

**

**Supplemental Figure 1.** Flow diagram of patient selection.

**
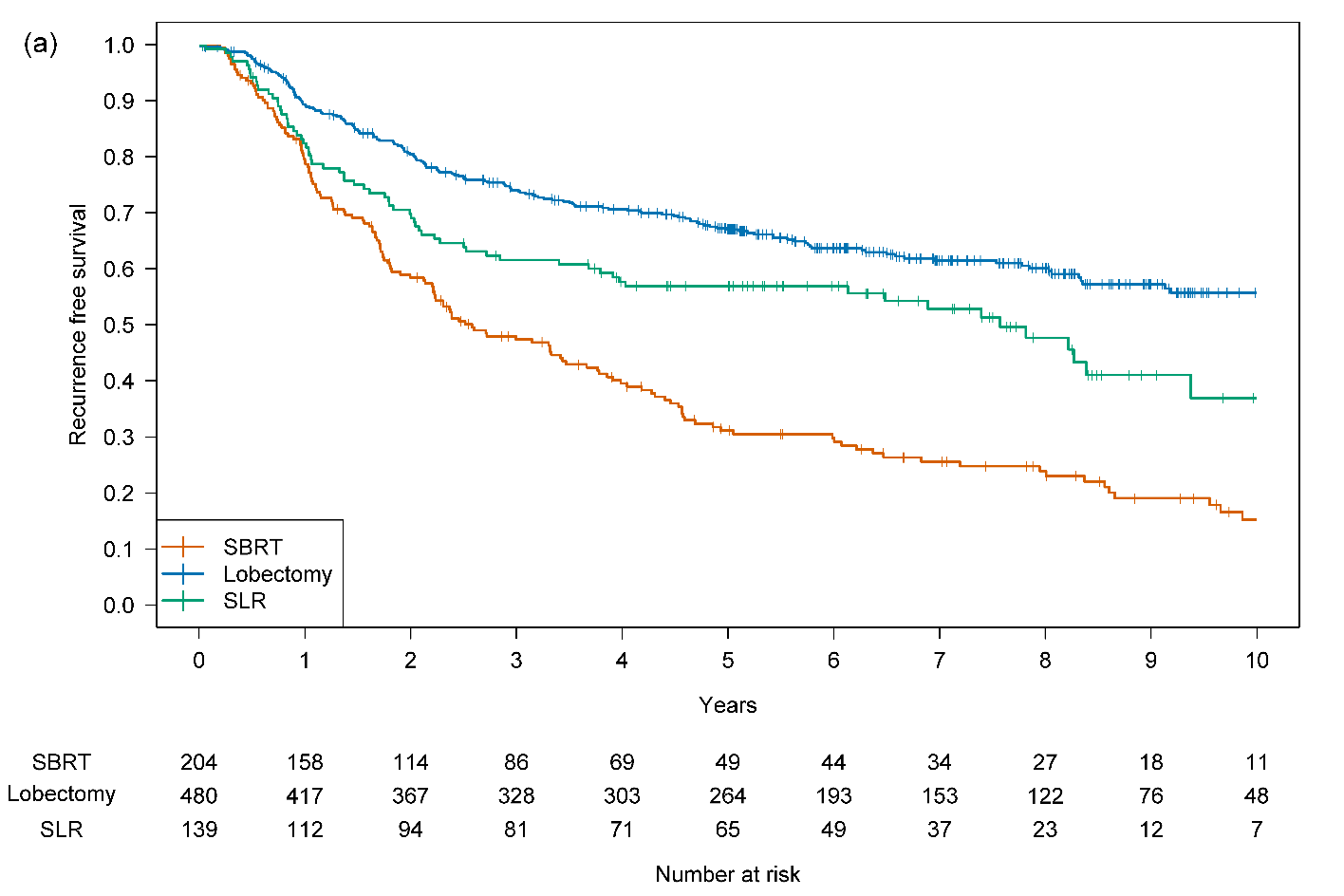

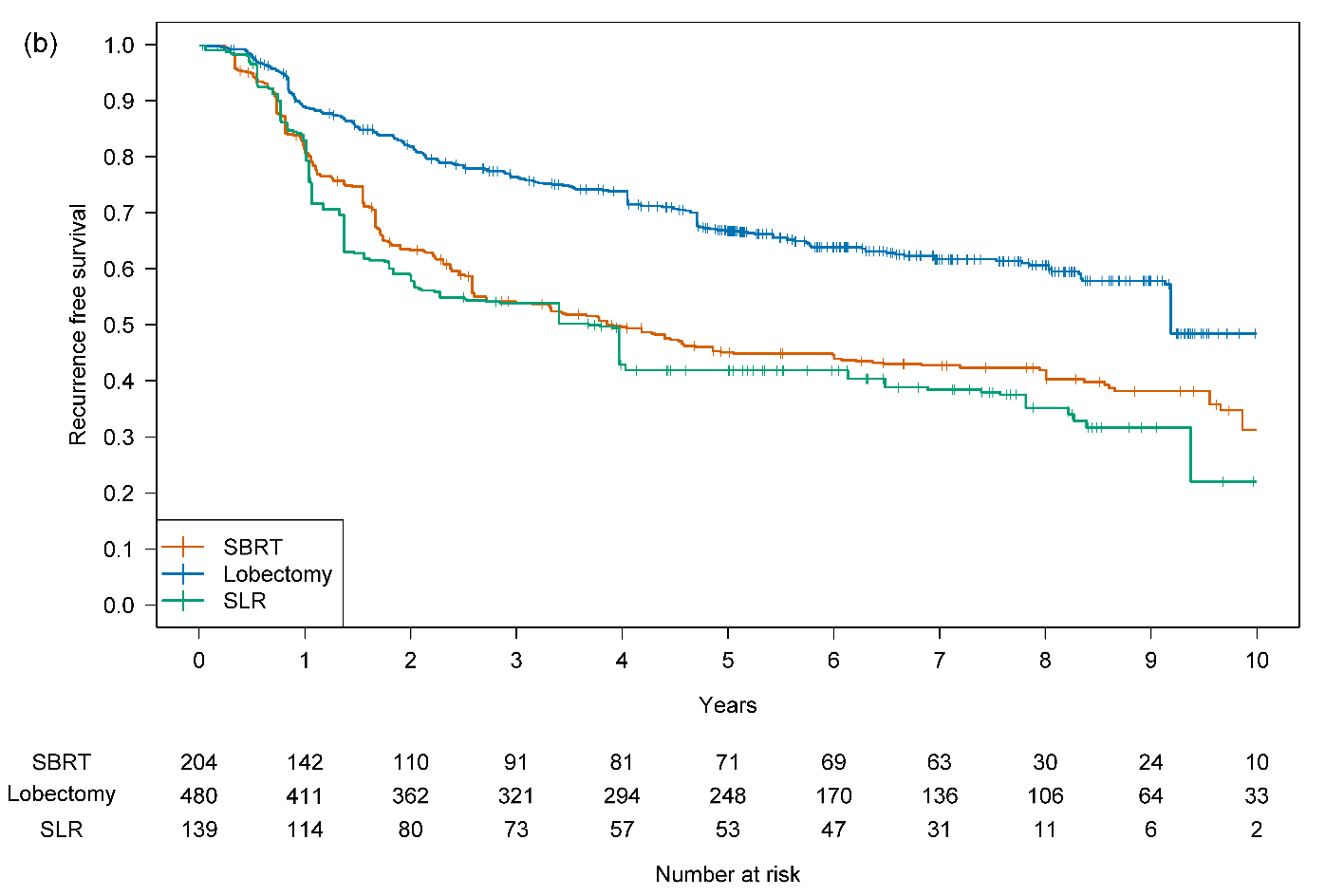
**

**Supplemental Figure 2.** Recurrence-free survival (a) in the unweighted cohort and (b) in the propensity-score weighted cohort.


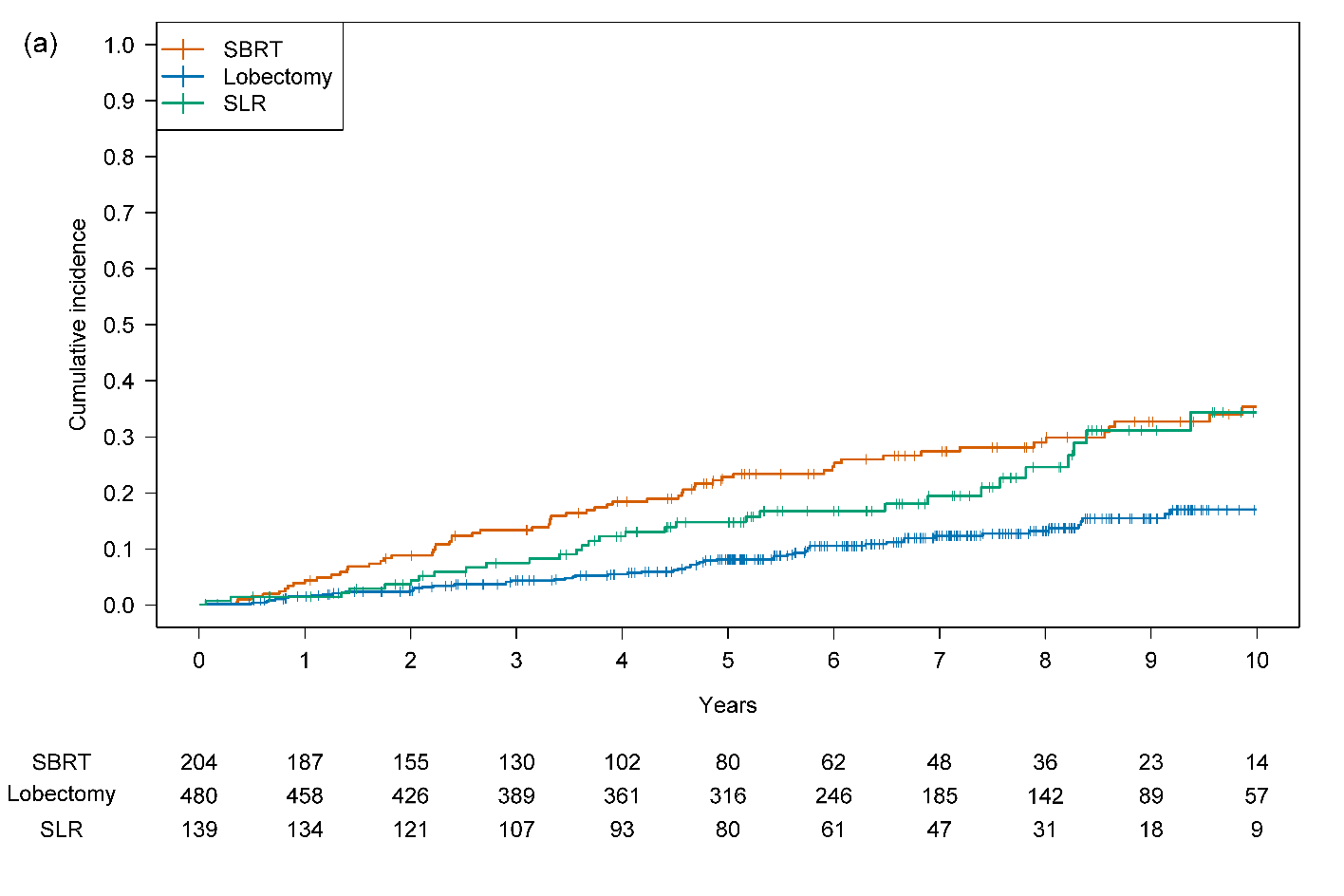

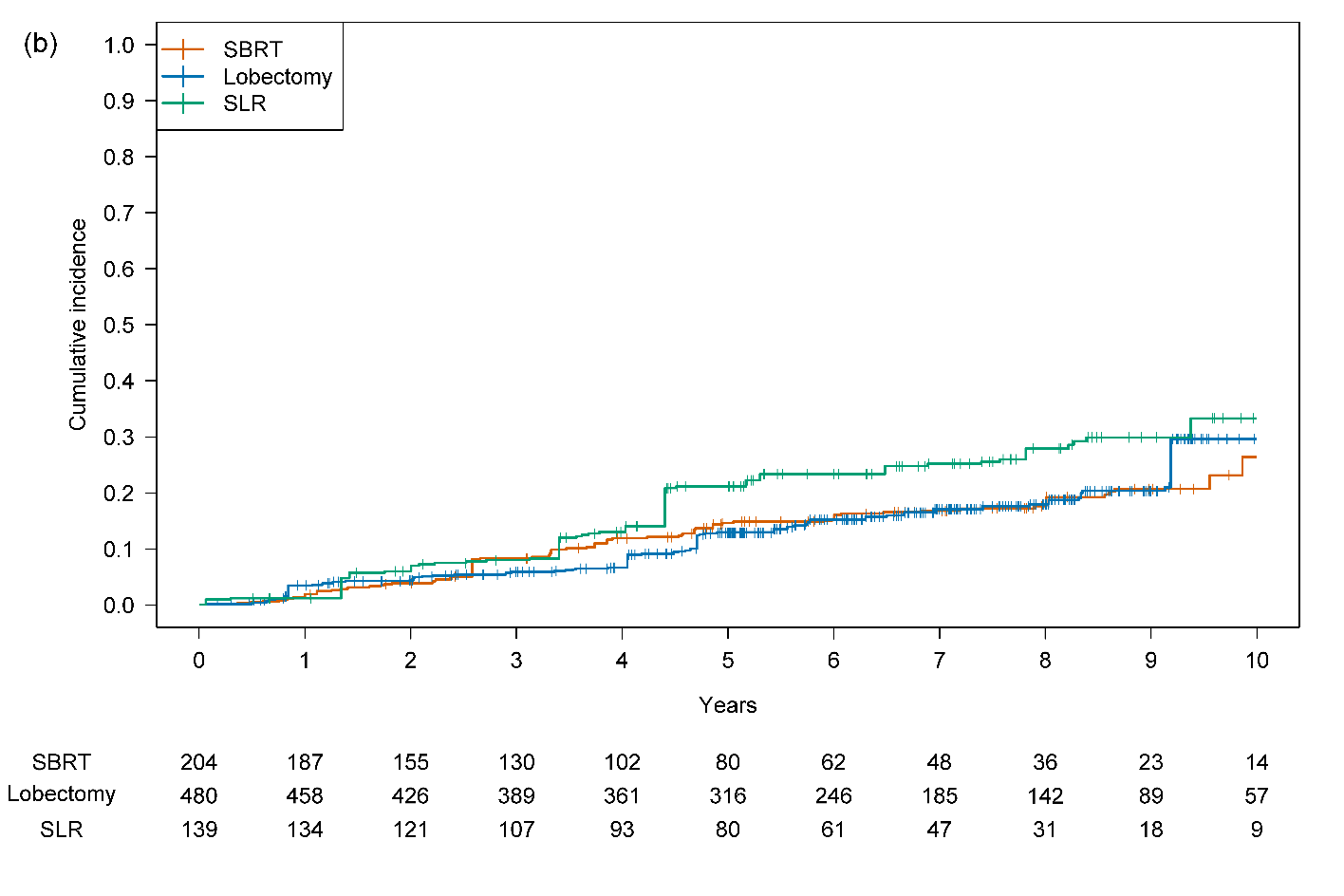


**Supplemental Figure 3.** Cumulative incidence rates of non-lung cancer death (a) in the unweighted cohort and (b) in the propensity score-weighted cohort


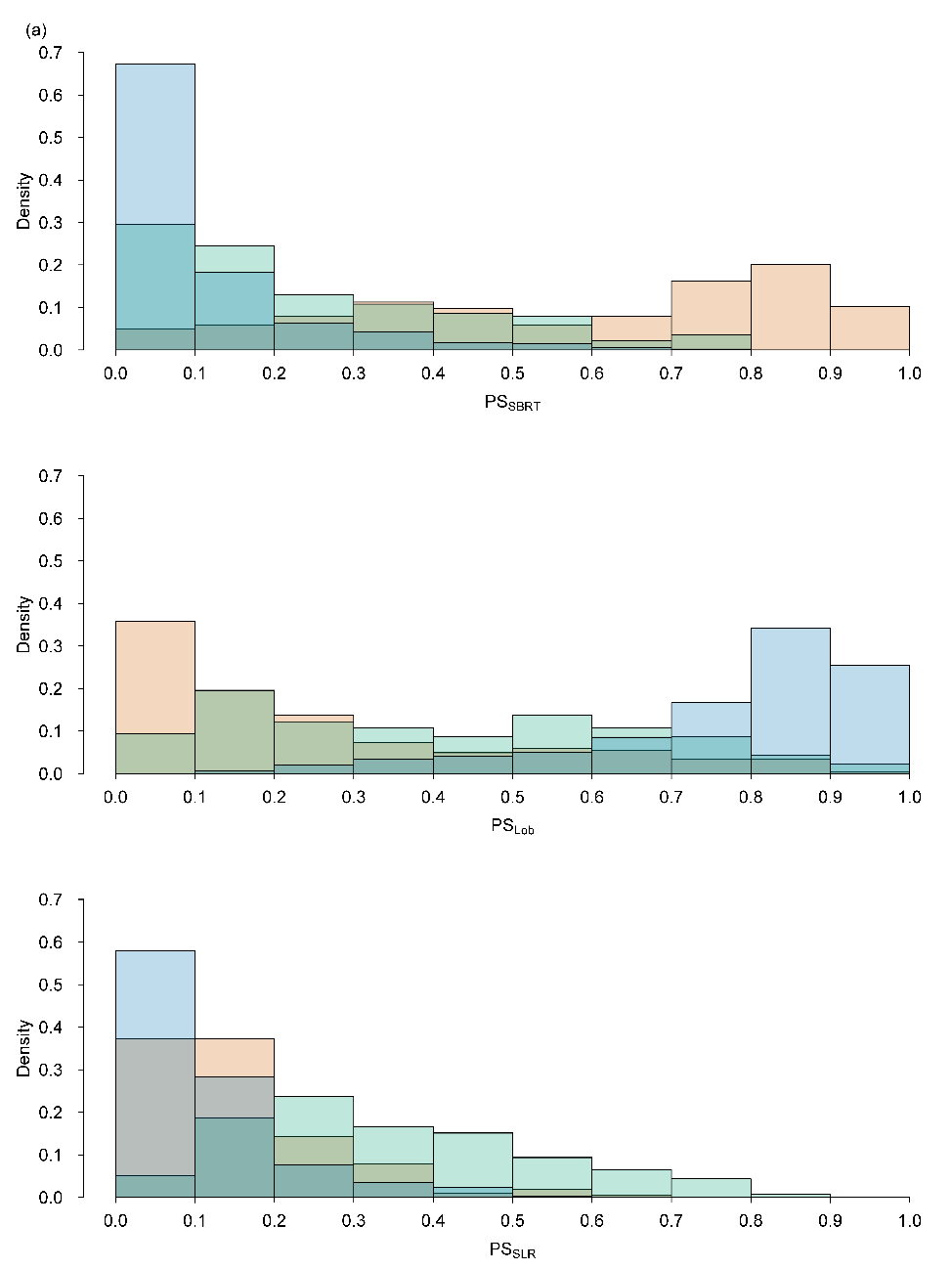

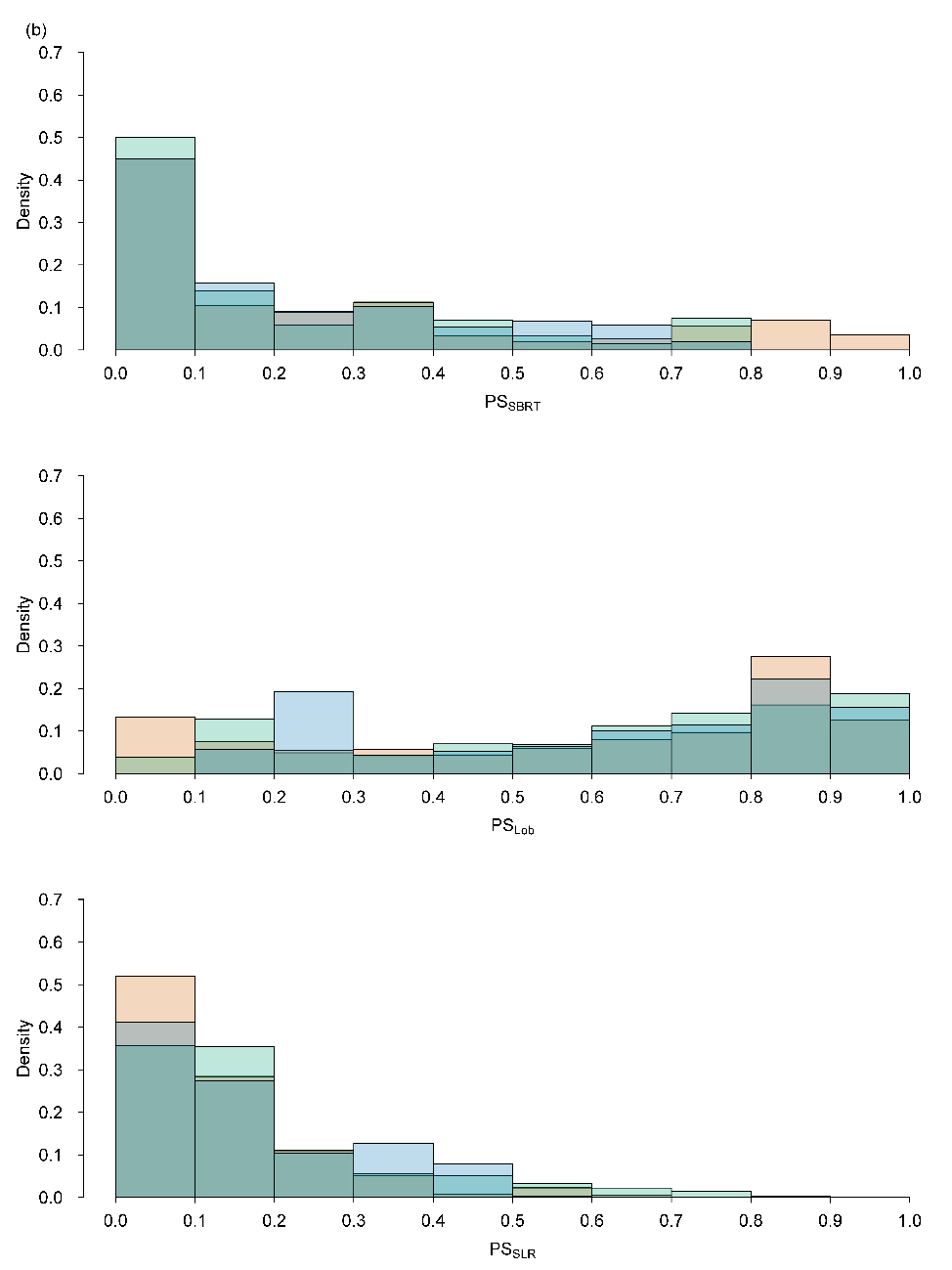


**Supplemental Figure 4.** The distributions of PS_SBRT_, PS_Lob_, and PS_SLR_ of (a) the unweighted cohort and (b) the weighted cohort. Each histogram indicates the density, and its color indicates the selected treatment (vermillion for SBRT, blue for lobectomy, and green for SLR). *Abbreviations:* PS_SBRT_, propensity scores for SBRT; PS_Lob_, propensity scores for lobectomy; PS_SLR_, propensity scores for SLR. The other abbreviations are the same as described in Table 1.

**Supplemental Table 1.** Cumulative incidence rate of non-lung cancer death and recurrence in stereotactic body radiotherapy (SBRT), lobectomy (Lob) and sublobar resection (SLR).

|  |  | **Unweighted cohort** | | | **Weighted cohort** | | | |
| --- | --- | --- | --- | --- | --- | --- | --- | --- |
|  |  | **SBRT** | **Lob** | **SLR** | | **SBRT** | **Lob** | **SLR** |
| **Non-lung cancer death** |  |  |  |  | |  |  |  |
| Event of interest | 5-year (%)  sHR [97.5% CI]  P value | 22.8%  (*reference*) | 8.2%  0.32 [0.22–0.48]  P < 0.001 | 14.8%  0.59 [0.36–0.98]  P = 0.019 | | 14.6%  (*reference*) | 13.0%  0.91 [0.42–1.97]  P = 0.78 | 21.1%  1.33 [0.48–3.68]  P = 0.53 |
| Competing event | 5-year (%)  sHR [97.5% CI]  P value | 30.6%  (*reference*) | 11.7%  0.29 [0.21–0.40]  P < 0.001 | 13.3%  0.30 [0.18–0.49]  P < 0.001 | | 25.3%  (*reference*) | 9.5%  0.33 [0.17–0.66]  P < 0.001 | 17.7%  0.61 [0.23–1.62]  P = 0.25 |
| **Local recurrence** |  |  |  |  | |  |  |  |
| Event of interest | 5-year (%)  sHR [97.5% CI]  P value | 36.9%  (*reference*) | 2.2%  0.05 [0.03–0.12]  P < 0.001 | 19.4%  0.55 [0.32–0.93]  P = 0.011 | | 29.1%  (*reference*) | 1.6%  0.05 [0.02–0.13]  P < 0.001 | 21.6%  0.87 [0.33–2.28]  P = 0.74 |
| Competing event | 5-year (%)  sHR [97.5% CI]  P value | 4.4%  (*reference*) | 5.2%  0.82 [0.39–1.71]  P = 0.54 | 5.0%  1.12 [0.45–2.82]  P = 0.78 | | 5.2%  (*reference*) | 8.6%  1.75 [0.43–7.21]  P = 0.37 | 4.8%  1.13 [0.23–5.71]  P = 0.86 |
| **Regional recurrence** |  |  |  |  | |  |  |  |
| Event of interest | 5-year (%)  sHR [97.5% CI]  P value | 23.5%  (*reference*) | 16.5%  0.73 [0.46–1.17]  P = 0.14 | 18.3%  0.89 [0.49–1.59]  P = 0.65 | | 22.5%  (*reference*) | 13.4%  0.64 [0.27–1.48]  P = 0.23 | 22.4%  1.33 [0.44–4.00]  P = 0.56 |
| Competing event | 5-year (%)  sHR [97.5% CI]  P value | 5.1%  (*reference*) | 4.6%  0.76 [0.37–1.56]  P = 0.40 | 4.2%  0.96 [0.38–2.42]  P = 0.92 | | 5.5%  (*reference*) | 8.1%  1.72 [0.43–6.86]  P = 0.38 | 4.0%  0.95 [0.19–4.80]  P = 0.95 |
| **Distant recurrence** |  |  |  |  | |  |  |  |
| Event of interest | 5-year (%)  sHR [97.5% CI]  P value | 35.6%  (*reference*) | 22.2%  0.60 [0.42–0.85]  P = 0.001 | 27.0%  0.74 [0.46–1.19]  P = 0.15 | | 37.6%  (*reference*) | 17.8%  0.48 [0.26–0.87]  P = 0.006 | 36.5%  1.25 [0.57–2.73]  P = 0.52 |
| Competing event | 5-year (%)  sHR [97.5% CI]  P value | 4.3%  (*reference*) | 4.0%  0.77 [0.37–1.64]  P = 0.44 | 4.1%  1.12 [0.44–2.67]  P = 0.78 | | 5.8%  (*reference*) | 7.4%  1.54 [0.37–6.37]  P = 0.49 | 3.8%  1.00 [0.20–4.95]  P = 1.00 |

*Abbreviations:* CI, confidence interval; sHR, subdistriibution hazard ratio. Other abbreviations are the same as Table 1.

**Supplemental Table 2.** Patient characteristics according to the treatment-oriented subgroups.

|  | **Lobectomy-oriented** | | | |  | **SLR-oriented** | | | |
| --- | --- | --- | --- | --- | --- | --- | --- | --- | --- |
|  | **SBRT** (n = 15) | **Lob** (n = 370) | **SLR** (n = 22) | ***P* value** |  | **SBRT** (n = 16) | **Lob** (n = 23) | **SLR** (n = 62) | ***P* value** |
| Age [y] | 72.7 (4.0) | 71.4 (4.2) | 72.6 (3.5) | 0.24 |  | 76.3 (5.0) | 76.5 (4.4) | 76.6 (5.0) | 0.96 |
| Sex (Male/Female) | 11/4 | 196/174 | 16/6 | 0.07 |  | 14/2 | 18/5 | 33/29 | 0.011 |
| ECOG-PS (0/1) | 15/0 | 368/2 | 22/0 | 0.90 |  | 9/7 | 4/19 | 38/24 | 0.13 |
| Smoking status (Current/Former/Never) | 3/8/4 | 96/109/165 | 8/7/7 | 0.23 |  | 3/13/0 | 9/10/4 | 14/28/20 | 0.019 |
| BMI (Under/Normal/Overweight) | 0/14/1 | 32/263/75 | 1/13/8 | 0.12 |  | 2/8/6 | 1/16/6 | 7/41/14 | 0.61 |
| CCI (0/1–2/3+) | 10/5/0 | 196/138/36 | 8/11/3 | 0.35 |  | 1/8/7 | 1/14/8 | 11/27/24 | 0.38 |
| FEV1 [L] | 2.15 (0.41) | 2.22 (0.56) | 2.39 (0.55) | 0.34 |  | 1.83 (0.42) | 2.07 (0.48) | 1.77 (0.42) | 0.020 |
| Tumor diameter [mm] | 26.1 (6.9) | 27.1 (8.9) | 26.0 (7.0) | 0.76 |  | 15.6 (4.5) | 15.4 (3.8) | 15.1 (4.3) | 0.90 |
| C/T ratio | 0.95 (0.15) | 0.82 (0.33) | 0.91 (0.21) | 0.16 |  | 0.88 (0.34) | 0.96 (0.21) | 0.88 (0.33) | 0.54 |
|  |  | | | |  |  | | | |
|  | **SBRT-oriented** | | | |  | **Treatment-neutral** | | | |
|  | **SBRT** (n = 122) | **Lob** (n = 10) | **SLR** (n = 18) | ***P* value** |  | **SBRT** (n = 51) | **Lob** (n = 77) | **SLR** (n = 37) | ***P* value** |
| Age [y] | 80.2 (5.7) | 81.0 (4.9) | 78.5 (4.9) | 0.43 |  | 76.0 (4.6) | 76.1 (4.6) | 75.2 (5.6) | 0.66 |
| Sex (Male/Female) | 94/28 | 7/3 | 12/6 | 0.59 |  | 33/18 | 53/24 | 27/10 | 0.71 |
| ECOG-PS (0/1) | 52/70 | 4/6 | 8/10 | 0.97 |  | 37/14 | 58/19 | 8/29 | 0.82 |
| Smoking status (Current/Former/Never) | 7/98/17 | 0/8/2 | 2/15/1 | 0.63 |  | 4/34/13 | 16/45/16 | 10/19/8 | 0.20 |
| BMI (Under/Normal/Overweight) | 26/77/19 | 1/8/1 | 1/14/3 | 0.47 |  | 9/35/6 | 12/52/13 | 6/24/7 | 0.92 |
| CCI (0/1–2/3+) | 13/68/41 | 3/5/2 | 1/12/5 | 0.32 |  | 8/27/16 | 14/41/22 | 7/21/9 | 0.96 |
| FEV1 [L] | 1.38 (0.56) | 1.77 (0.52) | 1.34 (0.31) | 0.12 |  | 1.88 (0.46) | 1.89 (0.45) | 2.19 (0.42) | 0.002 |
| Tumor diameter [mm] | 24.4 (7.3) | 28.1 (10.9) | 27.8 (8.9) | 0.097 |  | 25.6 (9.4) | 25.7 (9.5) | 23.9 (8.9) | 0.66 |
| C/T ratio | 0.95 (0.21) | 0.90 (0.21) | 0.94 (0.24) | 0.77 |  | 0.91 (0.27) | 0.92 (0.24) | 0.80 (0.38) | 0.12 |

Data are presented as mean (standard deviation) for continuous variables and as numbers for categorical variables.

Abbreviations and the details of unavailable data are the same as described in Table 1.
